# OMRT-13. Delivery of Ubidecarenone (BPM 31510) to mitochondria effectuates metabolic reprogramming and redox activated apoptosis in Glioblastoma

**DOI:** 10.1093/noajnl/vdab070.037

**Published:** 2021-07-05

**Authors:** Rangaprasad Sarangarajan, Seema Nagpal, Jiaxin Sun, Anne Diers, Punit Shah, Vladimir Tolstikov, Gregory Miller, Vivek Vishnudas, Stephane Gesta, Michael Kiebish, Elder Granger, Niven Narain, Lawrence Recht

**Affiliations:** 1 BERG, Framingham, MA, USA; 2 Department of Neurology and Clinical Neurosciences, Stanford University, Palo Alto, CA, USA

## Abstract

GBM is a highly metabolic cancer phenotype that confers sustained growth and evasion of cell death mechanism via mitochondrial dysregulation. Efforts to re-engage mitochondrial metabolism via anti-cancer therapeutics has not been successful. BPM 31510 is a CoQ10-lipid conjugate nanodispersion for delivery of CoQ10 preferentially to mitochondria of human cells. BPM has demonstrated anti-cancer effects across multiple cancers, without adversely affecting normal tissue. The anti-cancer mechanism of CoQ10 was elucidated by Interrogative Biology, a data-driven approach to understand disease biology, identify targets and biomarkers of disease. Specifically, oncogenic and corresponding non-disease normal cell-based models (e.g. breast, liver, prostate, kidney) were subjected to cancer specific perturbations (e.g. hypoxia, metabolic stress). Comprehensive multi-omic (genome, proteome, lipidome, metabolome) and functional endpoints data were profiled. A Bayesian artificial intelligence analytics was used to generate network models in a data driven manner to identify BPM 31510 mechanism (i.e. shift in oxygen and glucose utilization, increase in oxidative stress and apoptosis in cancer cells). BPM 31510 re-capitulated its anti-cancer effect in GBM models, including LN-229 xenograft and C6 glioma allograft, both as monotherapy and in combination with temozolomide (TMZ)/radiation. The platform generated network maps from longitudinal pharmacodynamic samples (20 samples/28 days) collected from GBM patient refractory to TMZ/radiation/bevacizumab (Phase 1, NCT03020602, Stanford) identified alterations in intermediary metabolism as drivers of Progression Free Survival (PFS) and Overall Survival (OS) in response to BPM 31510 treatment. The platform supports the ongoing Phase 2 trial of adjuvant BPM 31510 plus TMZ/radiation in newly diagnosed GBM patients and potential accelerated approval.

